# Correspondence

**Published:** 1912-03

**Authors:** Nathan Strauss


*To the Editor*:

In the efforts that are being made to protect the babies from milk-borne diseases such as tuberculosis, typhoid, scarlet fever, diphtheria, etc., the well considered policy of the Public Health Service and of the foremost health officers of the country is seriously hindered by attacks based upon ignorance.

The statement is made that the use of pasteurized milk causes rickets, scurvey and anaemia.

In feeding 25,000 babies with pasteurized milk through my infant milk depots in New York City alone, never has one case of scurvey or rickets developed. Dr. Variot, in Paris, fed 13,000 babies on sterilized milk without causing these diseases.

In order to bring this issue to an end, I offer through you $1,000 for any case of scurvey or rickets or anaemia caused by feeding a baby with properly pasteurized milk.

If any such case is alleged in answer to this challenge, I will leave the determination of the facts to Dr. Rupert Blue, Surgeon General; Dr. M. J. Rosenau, Professor of Hygiene and Preventive Medicine at Harvard, and Dr. John F. Anderson, director of the Hygienic Laboratory at Washington, or any jury that they may choose.

Very sincerely yours,

NATHAN STRAUSS.

